# Expression of ATRX, DAXX, PDX1, ARX, and somatostatin receptors in pancreatic neuroendocrine tumors: a clinicopathological study

**DOI:** 10.3389/fendo.2026.1820433

**Published:** 2026-05-11

**Authors:** Oana A. Ciobanu, Carmen Sorina Martin, Simona Fica, Vlad Herlea, Irina Tudose, Florina Vasilescu, Irina Sandra, Carmen G. Barbu

**Affiliations:** 1 Endocrinology Department, Carol Davila University of Medicine and Pharmacy, Bucharest, Romania; 2 Endocrinology and Diabetes Mellitus, Nutrition and Metabolic Disorders Department, Elias Hospital, Bucharest, Romania; 3 Pathology Department, Fundeni Clinical Institute, Bucharest, Romania; 4 Pathology Department, Carol Davila University of Medicine and Pharmacy, Bucharest, Romania; 5 Pathology Department, Elias Hospital, Bucharest, Romania; 6 Pathology Department, Central Military University Emergency Hospital, Bucharest, Romania; 7 Oncology Department, Carol Davila University of Medicine and Pharmacy, Bucharest, Romania

**Keywords:** clinicopathologic features, immunohistochemistry, pancreatic neuroendocrine tumors, prognosis, tumor biomarkers

## Abstract

**Background:**

Recent studies have identified ATRX/DAXX and PDX1/ARX as biomarkers defining novel pancreatic neuroendocrine tumor (PanNET) subtypes, while the clinical significance of somatostatin receptors (SSTRs) remains incompletely understood.

**Methods:**

We retrospectively analyzed 58 surgically resected primary PanNET samples and performed immunohistochemical evaluation of ATRX, DAXX, ARX/PDX1, and SSTR2a/5. The primary goal was to assess associations between biomarker expression and clinicopathological parameters. Secondary analyses explored relationships with recurrence-free survival (RFS), cancer-specific survival (CSS), and overall survival (OS). Quantitative expression scores were calculated, receiver operating characteristic (ROC) curve analysis was performed, and survival outcomes were assessed using Kaplan–Meier analysis.

**Results:**

The loss of DAXX or ATRX expression (86.3%) was more commonly observed in nonfunctional PanNETs cases compared to the functional PanNET group (13.7%) (p = 0.02). SSTR5 positive tumors were associated with longer median OS (73 vs. 7 months, p < 0.001) and RFS (36 vs. 6 months, p = 0.005). However, the difference in OS was not confirmed by Kaplan–Meier analysis (log-rank p = 0.078). In PanNETs ≥ 2 cm, a tumor size cutoff of 2.45 cm predicted ATRX/DAXX mutations with 96.9% sensitivity and 75% specificity (AUC 0.922, p = 0.007), in a cohort of 36 patients.

**Conclusions:**

ATRX/DAXX loss was more prevalent in nonfunctional PanNETs (p = 0.02). Although SSTR5-positive tumors were associated with longer median OS, this difference was not confirmed by Kaplan–Meier analysis. Furthermore, tumor size demonstrates predictive value for ATRX/DAXX loss in PanNETs ≥2 cm, highlighting the relationship between tumor morphology and molecular alterations, however, this finding remains hypothesis-generating and requires further validation.

## 1 Introduction

Neuroendocrine neoplasms (NENs) are highly heterogeneous tumors that can develop in various parts of the body, including lung and gastro-entero-pancreatic (GEP) sites. The latest 2022 World Health Organization (WHO) classification aims to standardize their classification by stratifying them into two groups of NENs, irrespective of their primary location: well-differentiated neuroendocrine neoplasms or neuroendocrine tumors (NETs) and poorly differentiated neoplasms or neuroendocrine carcinomas (NECs) (1). The fivefold increase in the pancreatic NETs (PanNETs) incidence over several decades has led to them representing the second most common type of NENs after pancreatic adenocarcinoma (2–6). The spectrum of manifestations in PanNETs varies from an indolent course to aggressive behavior, as evidenced by the significant difference in the 5-year survival rates between localized disease (93%) and advanced disease (27%) (7, 8). Still, unlike other types of NENs, PanNETs are associated with a poorer prognosis due to their malignant potential, higher grades, or advanced disease (approximately 65%) upon diagnosis (2, 3, 9). Consequently, there is an unmet need to identify new molecular subtypes based on prognostic biomarkers in PanNETs due to the restricted utility of clinicopathological markers in clinical practice. For example, intratumoral heterogeneity, sampling issues, and interpretation errors can affect the mitotic index and Ki-67 measurements, particularly in biopsy samples (10, 11). Moreover, patients with WHO grade 1 PanNETs can develop distant metastases (12, 13)), and the TNM (tumor, node, metastasis) stage is not always predictive (14–16). Additionally, extensive database studies and meta-analyses have not confirmed the tumor size as an independent prognostic factor, although it is considered an essential presurgical marker (10, 17, 18).

A key characteristic of most NENs is the overexpression of somatostatin (SST) receptors (SSTRs), particularly SSTR2 and SSTR5. By leveraging SSTR expression, imaging modalities can be employed to evaluate the primary tumor location, as well as the presence and extent of metastases (11). Furthermore, detecting these receptors enables the identification of target cells for the assessment of potential therapeutic targets, such as somatostatin analogs (SSAs) or peptide receptor radionuclide therapy (PRRT). An immunohistochemistry (IHC) analysis is a cost-effective and reliable method for detecting SSTR2 and SSTR5 in NENs. Although SSTR2 and SSTR5 positivity may correlate with improved survival in GEP-NENs, few studies have used IHC to precisely localize and quantify SSTR2 and 5 expressing cells in PanNETs. Furthermore, the differential expression patterns of SSTR subtypes and their association with clinico-pathological characteristics for risk stratification in PanNETs, as well as the overall pathological significance of SSTR expression, remain unclear (19, 20).

Recent studies have highlighted the significant correlation between a poor prognosis in PanNETs and the activation of alternative telomere lengthening (ALT), driven by mutations in the Death domain-associated protein (DAXX) or Alpha-thalassemia X-linked intellectual disability chromatin remodeler (ATRX) genes (12, 21–24). These mutually exclusive mutations lead to epigenetic progression, increase the risk of recurrence after surgery, and can be associated with ALT (22, 25). Mutations in both ATRX and DAXX lead to the subsequent loss of nuclear expression of their proteins, which can be assessed through IHC (26). PanNETs are characterized by a distinct genetic profile compared to pancreatic ductal adenocarcinoma, with MEN1 being the most frequent mutation, followed by ATRX or DAXX mutations (in approximately 40% of cases), and alterations in the mTOR pathway. While most PanNETS are sporadic, a variable proportion occur in hereditary syndromes such as multiple endocrine neoplasia type 1 (MEN 1), Von Hippel-Lindau (VHL) and neurofibromatosis type 1 (NF1) (27). Hereditary PanNETs patients benefit from routine genetic testing and typically exhibit a more indolent course, impacting prognosis and management. Still, recent studies also identified germline mutations in genes such as MUTYH, CHEK2, BRCA2, MEN1, and VHL in approximately 17% of PanNET patients (28).

Although the “watch and wait” strategy is common for cases of small tumors, 15% of PanNETs under 2 cm have lymph node metastases (12, 29, 30), a concern for decisions about size-based surveillance versus surgery (25). In this ongoing debate, the assessment of ATRX/DAXX status can be clinically valuable in managing small PanNETs, especially in patients with genetic syndromes such as MEN1, due to its demonstrated prognostic significance.

The ARX (aristaless-related homeobox gene) and PDX1 (pancreatic and duodenal homeobox 1) transcription factors regulate alpha (α) and beta (β) cell differentiation, and are associated with ATRX/DAXX/MEN1 (ADM) status. Based on these patterns, PanNETs can be classified into α-like (MEN1-mutated, favorable prognosis), β-like (ADM wild-type (WT), less aggressive), and intermediate (DAXX/ATRX-mutated, associated with ALT activation) groups (31). Immunohistochemically, strong ARX expression characterizes α-like and intermediate tumors, whereas PDX1 positivity defines β-like tumors. Therefore, ARX/PDX1 IHC supports the identification of cell lineage in PanNETs (26, 32, 33).

According to a meta-analysis (34), ATRX/DAXX could serve as indicators for determining the clinical course of the disease and the survival endpoints, although integrating ARX/PDX1 or linking SSTR subtype expression to survival is still debated (35). While the IHC of these markers could cost-effectively supplement established prognostic biomarkers, their applicability to Romanian PanNET patients remains an open question. Therefore, the primary purpose of our study was to perform a comprehensive analysis of the protein expression at the time of diagnosis, evaluated by immunolabeling for ARX/PDX1 and ATRX/DAXX, as well as for SSTR2a and 5 with standard clinicopathological factors in cases of primary PanNENs. Furthermore, our secondary objective was to evaluate the prognostic significance of ARX/PDX1, ATRX/DAXX, and SSTR2a/5 concerning the clinical outcome of PanNET patients.

## 2 Materials and methods

The current study was an observational study conducted during manuscript preparation in accordance with the REMARK (36) and STROBE (37) guidelines. The case selection design is presented in **Table 1**.

**Table 1:** Flow-chart of the study.

Patients, treatment and variables study and the marker	Remarks		
Categorial binary marker	M1 ATRX = 0 negative, 1 positive M2 DAXX = 0 negative, 1 positive M3 ARX = 0 negative, 1 positive M4 PDX1 = 0 negative, 1 positive M5 SSTR 2a = 0 negative, 1 positive M6 SSTR 5 = 0 negative, 1 positive		
Further variables	V1=age, V2=sex, V3=grade, V4=Ki67 index, V5=size, V6= secretion; V7= primary tumors, V8= other non-endocrine neoplasms V9=pT classification, V10= pN (lymph node invasion), V11= perineural invasion, V12= lymphovascular invasion, V13 =necrosis V14= metastasis, V15= distant synchronous metasases, V16= distant metachronous metastases, V17= tumor location, V18= tumor secretion, V19=tumor genetics, V20= number of metastases, V21= locoregional recurrence, V22 = resection status, V23=overall survival, V24= recurrence free survival, V25= cancer specific survival, V26= treatment with SSAs, V27= tumor size prior to surgery (<2 cm; ≥2 cm), V28= score of ATRX expression, V29 = score of DAXX expression, V30= score of ARX expression, V31= score of PDX1 expression, V32= score of SSTR2a expression, V33= score of SSTR5 expression.		
Patients	N	Remarks	
Assessed for eligibility	93	Disease: PanNETs, grade G1, G2 Patients source: 2005-2023, “Elias and Central Military “Dr. Carol Davila” University Hospitals and “Fundeni” Clinical Institute Sample source: archived specimens available.	
Excluded	3	Only fine needle aspiration biopsies (FNAB) available	
	17	Patients who did not attend two clinic visits with at least 3 months of follow-up between them.	
Included	15	High proportion of missing critical clinical or pathologic data	
	58	Patients aged ≥ 18 years Well-differentiated, grades G1-G2 NETs Previously untreated with SSA	
With outcome events	58	Overall survival (OS): death from any cause (58 patients, 8 events) Recurrence free survival (RFS): local or system recurrence or death from any cause (58 patients, 28 events) Cancer specific survival (CSS): death from PanNET: (58 patients, 4 events)	
Statistical analyses of survival outcomes	Patients	Events	Variables
Analysis			
A1: Univariable	58	8 (OS); 28 (RFS); 4 (CSS)	M, V1-V27
A2: Multivariable	58	8 (OS); 28 (RFS); 4 (CSS)	M, V1-V27

### 2.1 Patients

A total of 58 patients with a diagnosis of PanNENs from January 2005 to December 2023 were recruited from the archives of the “Elias” Emergency University Hospital, the Central Military Emergency Hospital “Dr. Carol Davila, and the “Fundeni” Clinical Institute. The inclusion criteria were: (1) the presence of formalin-fixed paraffin-embedded (FFPE) blocks obtained from surgical specimens of well-differentiated, grades G1-G2 NETs within the pancreas as per the WHO 2010 classification; (2) cases with sufficient material for ancillary studies; (3) surveillance and survival data; (4) an age of 18 years or older; and (5) written consent for the use of the samples for research purposes. The exclusion criteria were (1) pancreatic neuroendocrine carcinomas, (2) only fine needle aspiration biopsy (FNAB) specimens, (3) a high proportion of missing critical clinical or pathologic data, and (4) patients lost to follow-up within less than 3 months. After reviewing each case, a database was created including 58 Pan-NETs with matched demographic and relevant clinical data, the available FFPE blocks, an imaging evaluation and/or pathological confirmation of disease recurrences or metastasis evaluations, follow-up data, the recurrence-free (RFS), cancer-specific (CSS), and overall survival (OS). Practical considerations, including the availability of tumor samples and cost constraints determined the sample size. For cases with multiple tumors, the largest tumor was measured; the tumor with the highest grade was also analyzed.

This study was approved by the Ethics Committees of the Elias University Emergency Hospital (approval number 123022024–1 on 23rd February 2024), the Central Military Emergency Hospital “Dr. Carol Davila” (approval number 680 on 29th February 2024), and the Fundeni Clinical Institute (approval number 66033 on 15th December 2023), where the IHC analyses were conducted. All the individuals involved in this study signed the informed consent agreement with the Helsinki Declaration.

### 2.2 Immunohistochemistry

Archival samples were stored for up to 18 years. All the FFPE blocks were collected for immunohistochemical investigations using the biotin-streptavidin method (38). FFPE tissue sections were stored at room temperature until further analysis. IHC was performed to confirm the neuroendocrine nature of the tumor according to the WHO 2022 definitions. A combination of two or more of the following immunohistochemistry markers were used for this purpose: chromogranin A, neuron specific enolase (NSE), synaptophysin (SYN). The following antibodies on 4 µm thick sections cut from the paraffin-embedded tissue were assessed on whole sections following the test manufacturer’s protocol: anti-chromogranin A (clone LK2H10), anti-synaptophysin (EP158), Ki-67 (SP6), anti-neuron specific enolase (NSE) (polyclonal), anti-SSTR2 (PA3-109), anti-SSTR5 (PA-41496), anti- ATRX (D1N2E), anti- DAXX (CL3580), anti-ARX (NBP2-80506), anti PDX1 (EP139), anti-SSTR2 (PA3-109), and anti-SSTR5 (PA1-41496). One pathologist evaluated the immunohistochemical stains while blinded to any patient data, including the outcome.

### 2.3 Outcomes

The primary outcome was the concordance between each marker ATRX, DAXX, ARX, PDX1, and SSTR2a and 5, as well as the combination of all of the following markers ATRX/DAXX, ARX/PDX1. It was evaluated using IHC with standard clinicopathological factors indicative of tumor aggressiveness in cases of primary PanNETs.

The secondary outcome was the prognostic significance of ARX/PDX1, ATRX/DAXX, and SSTR2a and 5 related to the clinical outcome of PanNET patients.

The clinical outcomes were assessed through RFS, CSS, and OS, as follows.

The OS was measured in months from the date of the surgery to the date of death from any cause (locoregional recurrences, distant metastases and second primary cancer were ignored).

The RFS was measured in months from the date of the surgery to the date of local or system recurrence or death from any cause, and second primary cancer was ignored.

The CSS was measured from the date of the surgery to the date of death from PanNET. The observations were censored in cases of death due to causes other than NEN-P; locoregional recurrences, distant metastases, and second primary cancer were ignored.

Patients who did not experience the event of interest at their last evaluation by the end of the analysis were censored.

### 2.4 Definitions

Ki-67 IHC was performed in the “hot spot” areas. At least 500 tumor nuclei or 2000 cells were examined to determine the average Ki-67 percentage. The average mitotic count in a 2mm^2^ area was noted. The higher value between the mitotic count and Ki-67 labelling was considered for WHO grading. All cases were graded according to the recent WHO 2022 classification.

The immunohistochemical expression of ATRX, DAXX, ARX and PDX1 was determined using the following scale: 0=negative nuclear staining (0-5% of cells are stained); 1+=week-positivity nuclear staining (5-10% of cells are stained); 2+=moderate-positivity nuclear staining (10-50% of cells are stained); 3+=strong-positivity nuclear staining (over 50% of cells are stained). The percentage of positive cells was established by analyzing 500–1000 cells and represents the ratio between the numbers of positive cells and the total number of analyzed cells. Surrounding islets in the normal tissue of the pancreas served as a positive internal control (lymphocytes and endothelial cells). Cytoplasmic staining was considered nonspecific and disregarded. For the subsequent statistical analysis, cases with heterogeneous staining were scored as a loss or negative staining. For cases interpreted as 1+, 2+, or 3+, the scores were considered positive in immunolabeling for ATRX and DAXX (12, 22), while tumor tissue with ≤5% nuclear expression was classified as negative, as previously described. However, for the evaluation of ARX and PDX1, only cases classified as 2 and 3 were considered positive, whereas scores of 0 or 1 were considered negative (12, 39, 40).

When evaluating SSTR2 and SSTR5 immunopositivity, we used the scoring system proposed by Keaemmerer et al. (41). The expression of SSTR2a and SSTR5 was evaluated based on both the proportion of tumor cells exhibiting positivity and the intensity of staining. The percentage of positive tumor cells was scored as follows: 0 for no positive cells; 1 for up to 10%; 2 for 10–50%; 3 for 51–80%; and 4 for 80% or more. The staining intensity was graded on a scale: 0 (no staining), 1 (mild reaction), 2 (moderate reaction), and 3 (strong reaction). The immunoreactivity score (IRS) was calculated by multiplying the percentage score by the staining intensity, resulting in a score between 0 and 12.

The distribution of IRS scores was classified as follows: scores of 0–1 were designated as negative, corresponding to a final score of 0; scores of 2–3 were considered indicative of weak positive expression, and were assigned a score of 1; scores ranging from 4 to 8 represented mild positive expression, with a score of 2; and scores of 9–12 reflected strong positive expression, and were assigned a score of 3. In the final evaluation, a score of 0 or 1 was interpreted as negative expression, whereas scores of 2, or 3 indicated positive expression, reflecting increasing immunoreactivity levels. The quantitative protein expression was evaluated using the IRS score (combined staining intensity and percentage score) for SSTR2a and SSTR5 (range 0–12). ATRX, DAXX, ARX, and PDX1 were scored semi-quantitatively (using a score from 0 to 3+) as previously described (12).Quantitative analysis of protein expression was performed to establish optimal cutoff values, utilizing the Youden Index to maximize sensitivity and specificity.

The tumor size (T), the presence of lymph node metastases (N), and the occurrence of distant metastases (M) were assessed according to the TNM classification staging AJCC version 9.

### 2.5 Statistical analysis

All statistical analyses were performed with SPSS (version 20), and p values <0.05 were considered statistically significant. Descriptive statistics of the patients’ clinical and qualitative data were expressed as numbers. The chi-square (*χ2)* analysis or Fisher’s exact test were used to compare groups and categorical data for the investigated clinicopathologic variables. The non-parametric Mann Whitney U test was used to compare continuous variables according to normality (Kolmogorov Smirnov test).

We conducted a ROC curve analysis to quantitatively assess the expression levels of the studied biomarkers. The optimal cutoff values for each marker, along with their corresponding sensitivity and specificity, were determined based on clinico-pathological parameters to evaluate and enhance their diagnostic performance. To enhance the robustness of the ROC analysis, a power analysis was conducted to ensure statistical reliability of the findings (42).

Survival curves were generated using the Kaplan–Meier method, and survival differences were determined using the log-rank statistics. A Cox regression analysis was employed to evaluate the prognostic significance of clinical and pathological features, with both univariate and multivariate analyses conducted using the enter method. No adjustment for multiple comparisons was performed.

## 3 Results

### 3.1 Patient characteristics

A total of 58 PanNETs that meet the inclusion criteria were identified. The patients’ characteristics and tumor features are summarized in **Table 2**. Across the entire cohort the median follow-up time was 16.5 months (range 3–259). The median age of the patients (34 females and 24 males) was 59 years (range 19–82 years). Tumors predominantly occurred in the body-tail region (n= 45, 77.6%). Fifty-four patients had sporadic PanNETs, while four were linked to MEN 1 syndrome. Among the study cohort, 22 patients had tumors smaller than 2 cm and underwent surgical intervention, whereas the majority (36 patients, 63.8%) presented with tumors ≥ 2 cm.

**Table 2:** Clinicopathological characteristics of the cohort of primary pancreatic neuroendocrine tumors.

Patients or tumor characteristics	Total, n=58
Age at diagnosis, median (range) (years)	59 (19, 82)	
Gender, n (%)		
Female	34 (58.6)	
Male	24 (41.4)	
Median tumor size (range), cm	2.5 (0.3, 9)	
Tumor location n (%)		
Head, neck and uncinate	13 (22.4)	
Body-tail	45 (77.6)	
Tumor secretion, n (%)		
Functional,	11 (19)	
Nonfunctional	47 (81)	
Tumor genetics, n (%)		
Sporadic	54 (93.1)	
Familial	4 (6.9)	
2022 WHO classification, n (%)		
G1	35 (60.3)	
G2	23 (39.7)	
G3	0 (0)	
Lymphovascular invasion, n (%)		
Absent	41 (70.7)	
Present	17 (29.3)	
Perineural invasion, n (%)		
Absent	52 (89.7)	
Present	6 (10.3)	
pTumor stage, n (%)		
T1	25 (43.1)	
T2	15 (25.9)	
T3	15 (25.9)	
T4	3 (5.2)	
Lymph node status (N), n (%)		
N0	46 (79.3)	
N1	12 (20.7)	
Metachronous metastases, n (%)		
Present	15 (25.9)	
Absent	43 (74.1)	
Synchronous metastases, n (%)		
Present	8 (13.8)	
Absent	50 (86.2)	
Number of metastases, n (%)		
0	38 (65.5)	
1	3 (5.2)	
2	5 (8.6)	
≥3	12 (20.7)	
Tumor size, n (%)		
<2cm	22 (36.2)	
≥ 2cm	36 (63.8)	
Surgery, n (%)		
R0	51 (87.9)	
R1	7 (12.1)	
Status at last follow-up, n (%)		
Alive	50 (86.2)	
Deceased	8 (13.8)	
Median follow up period (range), months	16.5 (3, 259)	

Results are reported as n (%) or median (min–max) as appropriate.

### 3.2 Tumor characteristics

The median tumor size was 2.5 cm (range 0.3–9cm). According to the 2022 WHO classification scheme, there were 35 G1 PanNETs and 23 G2 PanNETs. Twenty-five cases were classified as pT1, 15 as pT2, 15 as pT3, and 3 as pT4. Lymph node metastases were observed in 12 cases (20.7%), synchronous distant metastases were present in 8 cases (13.8%), and metachronous distant metastases were identified in 15 cases (25.9%).

### 3.3 Biomarkers status

**Figure 1:**
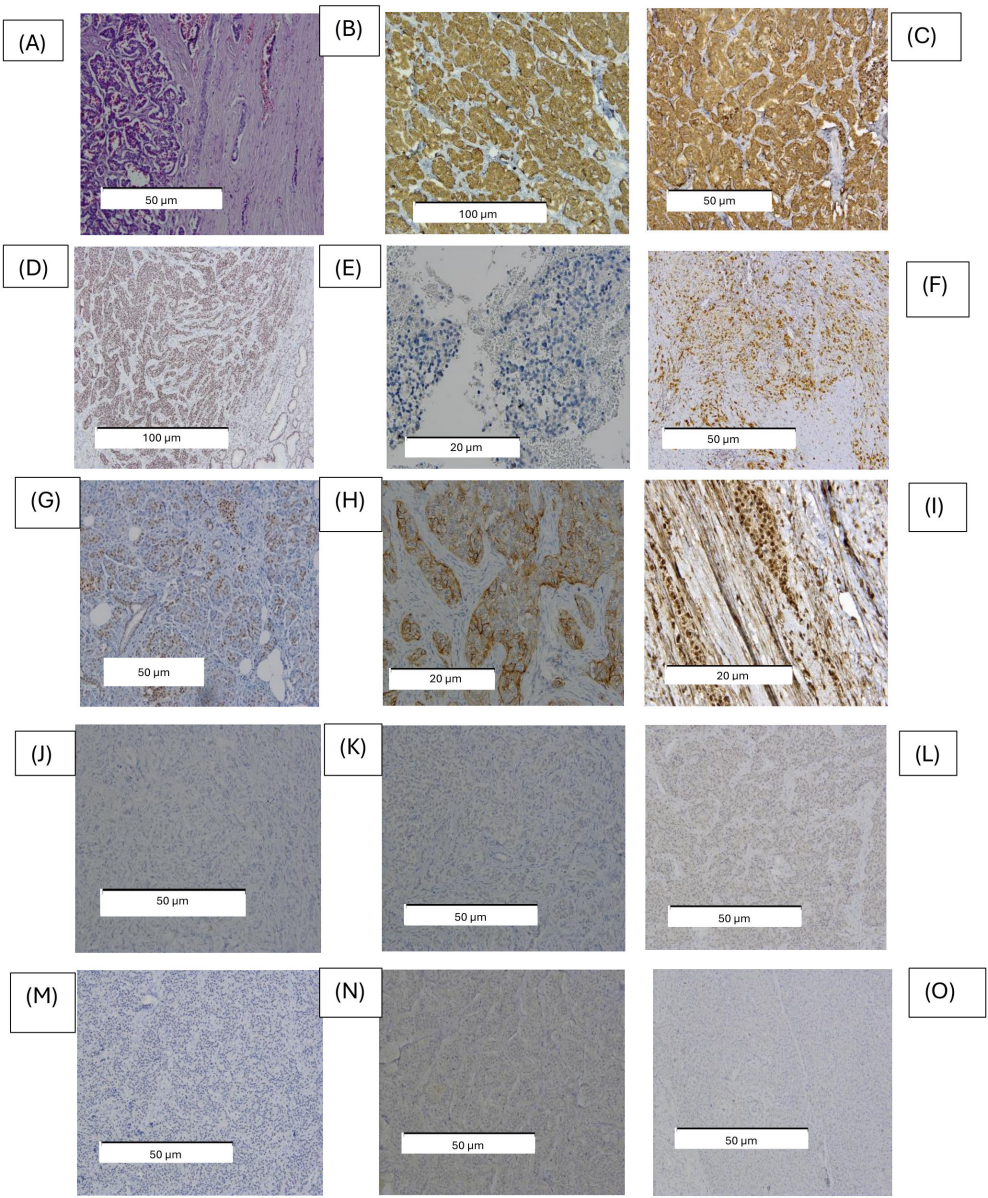
Representative examples of PanNETs evaluated through immunolabeling for the expressions of ATRX, DAXX, ARX, PDX1, and SSTR2a and SSTR5. Representative images of PanNETs with immunohistochemical staining **(a)** hematoxylin and eosin staining (100 x), **(b)** positive expression for SYN (100x), **(c)** CgA (200x), **(d)** positive expression for DAXX (100 x), **(e)** positive expression for ATRX (400 x), **(f)** positive expression for ARX (200 x), **(g)** positive expression for PDX1 (200 x) **(h)** positive expression for SSTR2 (400 x) **(i)** positive expression for SSTR5 (400 x) which exhibited preserved nuclear expression, **(j)** negative expression for ATRX (200 x); **(k)** negative expression for DAXX (200 x); **(l)** negative expression for ARX (200 x); **(m)** negative expression for PDX1 (200 x); **(n)** negative expression for SSTR2a (200 x); **(o)** negative expression for SSTR5 (200 x); ATRX, Alpha-thalassemia X-linked intellectual disability chromatin remodeler; CgA, chromogranin A; DAXX, death domain-associated protein; SYN, synaptophysin; ARX, aristaless-related homeobox gene; PDX1, pancreatic and duodenal homeobox 1, SSTR, somatostatin receptors (43).

In our PanNETs cohort (**Figure 1**), the overall positive rates of SSTR expression were 53.4% for SSTR2a (31 cases) and 8.6% for SSTR5 (5 cases). After excluding PanNETs without synchronous metastases, tumors with negative SSTR2a or 5 expression were more frequently associated with metachronous metastases compared to those with both positive expression (75% versus 25%, χ²=6.2, p = 0.038).

ATRX, DAXX, ARX and PDX1 expressions were evaluated both individually and in combination, with concurrent loss considered as a distinct molecular subgroup. We found the nuclear expression of ATRX in 11 samples (19%); respectively, DAXX was observed in 10 samples (17.2%). The loss of ATRX was noted in 47 samples (81%), while the loss of DAXX was found in 48 samples (82.8%). The nuclear expression of ARX was identified in 8 cases (13.8%) and that of PDX1 was found in 22 cases (37.9%). In the subgroup of nonfunctional (NF) PanNETs, we identified ARX+ tumors in 6 out of 46 cases (13%) and PDX1+ tumors in 15 out of 46 cases (32.6%). In the metastatic PanNETs subgroup, ARX- tumors accounted for 18 out of 20 cases (90%), while PDX1- tumors comprised 14 out of 20 cases (70%).

### 3.4 Single biomarkers

We analyzed our PanNETs cohort using both classical markers and newly proposed markers.

Lymph node invasion at diagnosis was significantly less frequent in G1 PanNETs compared to G2 PanNETs (25% vs. 75%; χ² = 7.899, p = 0.008). Furthermore, the proportion of patients with synchronous metastases was lower for G1 tumors compared to G2 PanNETs (25% versus 75%, χ²=4.845, p = 0.048). The tumors were further categorized based on the number of metastases, providing a comprehensive report. G1 tumors were most common in cases without metastases, representing 82.85% of such cases, compared to 5.71% for tumors with one metastasis, 2.85% for those with two metastases, and 8.57% for tumors with more than three metastases (p = 0.003 χ²= 13.766). Finally, 97.14% of G1 tumors and 73.91% of G2 tumors achieved R0 resection (χ² = 7.057, p = 0.013). Moreover, 95.45% of tumors measuring <2 cm demonstrated an N0 status, whereas 30.55% of tumors measuring ≥2 cm exhibited lymph node invasion at diagnosis (χ²=5.630, p = 0.021). In the same cohort, synchronous metastases were more prevalent in tumors measuring ≥2 cm compared to those <2 cm (100% versus 0%) (χ²=5.671, p = 0.019). Tumors measuring <2 cm were associated with a higher frequency of R0 resection margins during surgery compared to those with R0 resection margins that were ≥ 2 cm (100% versus 80.55%, χ²=4.865, p-value =0.037).

Additionally, SSTR2a-positive tumors were more common in PanNETs without lymphovascular invasion compared to SSTR2a-negative tumors (85.19% versus 58.06%, χ²=5.123, p = 0.041) (**Supplementary Table 1**). Furthermore, each newly proposed marker was individually assessed for its relevance to PanNETs regarding various clinicopathological variables and the established gold-standard prognostic markers used in daily clinical practice (**Table 3**).

**Table 3:** Clinical and pathological comparison of ATRX, DAXX, ARX, and PDX1 expressions in 58 primary pancreatic neuroendocrine tumors.

Clinicopathological characteristics	ATRX negative n= 47 (81%)	ATRX positive n=11 (19%)	P value	DAXX negative n= 48 (82.8%)	DAXX positive n= 10 (17.2%)	P value	ARX negative n=50 (86.2%)	ARX positive n= 8 (13.8%)	P value	PDX1 negative 36 (62.1%)	PDX1 positive 22 (37.9%)	P value
Age at diagnosis, (range), years	61 (22,82)	48 (19,73)	0.180	61 (22,82)	51.5 (19,72)	0.354	56 (19,82)	66 (46,72)	0.055	61.5 (22,82)	49.5 (19,78)	0.496
Gender, n (%)												
Female	27(57.45%)	7 (63.64%)	1.000	27 (56.25%)	7 (70%)	0.49	30 (60%)	4 (50%)	0.706	19 (52.78%)	15 (68.18%)	0.248
Male	20(42.55%)	4 (36.36%)		21 (43.75%)	3 (30%)		20 (40%)	4 (50%)		17 (47.22%)	7 (31.82%)	
Mean tumor size (range), cm	2.5 (0.3,9)	2.4 (0.8,6.2)	0.913	2.7 (0.3,9)	2.25 (0.8, 2.6)	0.187	2.5 (0.3,9)	2.45 (1.2,7.3)	0.304	2.8 (0.3, 7.3)	2.45 (0.4, 9)	0.422
Tumor location, n (%)												
Head, neck and uncinate	8 (17.02%)	5 (45.45%)	*0.042*	9 (18.75%)	4 (40%)	0.208	11 (22%)	2 (25%)	1.000	6 (16.67%)	7 (31.82%)	0.179
Body-tail	39 (82.98%)	6 (54.55%)		39 (81.25%)	6 (60%)		39 (78%)	6 (75%)		30 (83.33%)	15 (68.18%)	
Tumor secretion, n (%)												
Functional	6 (12.8%)	5 (45.5%)	*0.013*	7 (14.58%)	4 (40%)	0.83	9 (18%)	2 (25%)	0.639	4 (11.11%)	7 (31.82%)	0.083
Non-functional	41 (87.2%)	6 (54.5%)		41 (85.42%)	6 (60%)		41 (82%)	6 (75%)		32 (88.89%)	15 (68.18%)	
Tumor genetics, n (%)												
Sporadic	45 (95.74%)	9 (81.82%)	0.159	46 (95.83%)	8 (80%)	0.134	46 (92%)	8 (100%)	1.000	34 (94.44%)	20 (90.91%)	0.630
Familial	2 (4.26%)	2 (18.18%)		2 (4.17%)	2 (20%)		4 (8%)	0 (0%)		2 (5.56%)	2 (9.09%)	
WHO classification, n (%)												
G1	29 (61.70%)	6 (54.55%)	0.662	30 (62.5%)	5 (50%)	0.462	31 (62%)	4 (50%)	0.700	22 (61.11%)	13 (59.09%)	0.792
G2	18 (38.30%)	5 (45.45%)		18 (37.5%)	5 (50%)		19 (38%)	4 (50%)		14 (38.89%)	9 (40.91%)	
Lymphovascular invasion, n (%)												
Absent	33 (70.21%)	8 (72.73%)	1.000	32 (66.66%)	9 (90%)	0.253	37 (74%)	4 (50%)	0.216	25 (69.44%)	16 (72.73%)	0.790
Present	14(29.79%)	3 (27.27%)		16 (33.33%)	1(10%)		13 (26%)	4 (50%)		11(30.56%)	6 (27.27%)	
Perineural invasion, n (%)												
Absent	43 (91.49%)	9 (81.82%)	0.318	43 (89.58%)	9 (90%)	1.000	45 (90%)	7 (87.5%)	1.000	33 (91.67%)	19 (86.36%)	0.664
Present	4 (8.51%)	2 (18.18%)		5 (10.42%)	1 (10%)		5 (10%)	1 (12.5%)		3 (8.33%)	3 (13.64%)	
pTumor stage (pT) n (%)												
T1	20 (42.55%)	5 (45.45%)	0.861	21 (43.75%)	4 (40%)	1.000	22 (44%)	3 (37.5%)	1.000	15 (41.67%)	10 (45.45%)	0.777
T2-T3-T4	27 (57.45%)	6 (54.55%)		27 (56.25%)	6 (60%)		28 (56%)	5 (62.5%)		21 (58.33%)	12 (54.55%	
Lymph node status (pN), n (%)												
N0	36 (76.60%)	10 (90.91%)	0.429	38 (79.17%)	8 (80%)	1.000	40 (80%)	6 (75%)	0.665	28 (77.78%)	18 (81.82%)	1.000
N1	11 (23.40%)	1 (9.09%)		10 (20.83%)	2 (20%)		10 (20%)	2 (25%)		8 (22.22%)	4 (18.18%)	
Metachronous metastases, n (%)												
Present	12 (25.53%)	3 (27.27%)	1.000	12 (25%)	3 (30%)	0.708	13 (26%)	2 (25%)	1.000	10 (27.78%)	5 (22.73%)	0.670
Absent	35 (74.47%)	8 (72.73%)		36 (75%)	7 (70%)		37 (74%)	6 (75%)		26 (72.22%)	17 (77.27%)	
Synchronous metastases, n (%)												
Present	8 (17.02%)	0 (0%)	0.331	8 (16.67%)	0 (0%)	0.328	8 (16%)	0 (0%)	0.583	5 (13.89%)	3 (13.64%)	1.000
Absent	39 (82.98%)	11 (100%)		40 (83.33%)	10 (100%)		42 (84%)	8 (100%)		31 (86.11%)	19 (86.36%)	
Tumor size, n (%)												
<2cm	17(36.17%)	2 (18.18%)	0.310	16 (33.33%)	3 (30%)	1.000	20 (40%)	2 (25%)	0.697	13 (36.11%)	9 (40.91%)	0.715
≥ 2cm	30 (63.83%)	9 (81.82%)		32 (66.67%)	7 (70%)		30 (60%)	6 (75%)		23 (63.89%)	13 (59.09%)	
Surgery, n (%)												
R0	40(85.11%)	11(100%)	0.327	41 (85.42%)	10 (100%)	0.336	43 (86%)	8 (100%)	0.577	32 (88.89%)	19 (86.36%)	1.000
R1	7(14.89%)	0 (0%)		7(14.58%)	0 (0%)		7 (14%)	0 (0%)		4 (11.11%)	3 (13.64%)	
Locoregional recurrence, n (%)												
Absent	44 (93.62%)	9 (81.82%)	0.237	44 (91.67%)	9 (90%)	1.000	46 (92%)	7 (87.5%)	0.538	33 (91.67%)	20 (90.91%)	1.000
Present	3(6.38%)	2 (18.18%)		4 (8.33%)	1 (10%)		4 (8%)	1 (12.5%)		3 (8.33%)	2 (9.09%)	
Recurrence, n (%)												
Absent	24 (51.06%)	6 (54.55%)	0.835	23 (47.92%)	7 (70%)	0.204	26 (52%)	4 (50%)	1.000	17 (47.22%)	13 (59.09%)	0.380
Present	23 (48.94%)	5 (45.45%)		25 (52.08%)	3 (30%)		24 (48%)	4 (50%)		19 (52.78%)	9 (40.91%)	
Overall survival (OS), (range), months	16 (3,84)	19 (3,259)	0.351	16.5 (3,259)	15 (3,216)	0.621	9 (3,259)	36 (3,84)	0.120	21 (3,259)	4.5 (3,73)	*0.039*
Recurrence free survival (RFS), (range), months	9 (3,73)	19 (3,156)	0.211	7.5 (3,120)	14 (3,156)	0.306	6 (3,156)	19.5 (3,56)	0.162	13 (3,156)	4.5 (3, 73)	*0.082*

Results are reported as n (%) or median (min–max) as appropriate. p-values were determined using Pearson’s chi-square or Fisher’s exact test for categorical variables, and the Mann–Whitney U test for continuous variables. Statistically significant differences are highlighted in bold font.

The loss of ATRX expression was observed to be more common in the NF-PanNETs tumor group (41 out of 47, 87.2%) than in the F-PanNETs group (6 out of 47, 12.8%) (χ²=6.198, p = 0.013). Moreover, ATRX- tumors were more frequently found in the body-tail region (39 out of 47, 82.98%) compared to the head, neck, and uncinate regions (8 out of 47, 17.02%) (χ²=4.144, p = 0.042).

### 3.5 Combined biomarkers

We analyzed a panel of marker combinations within our cohort (**Table 4**). Our study cohort was categorized into four immunophenotypic groups based on ARX and PDX1 expression: ARX+PDX1- (4 cases, 6.9%), ARX-/PDX1+ (18 cases, 31%), ARX+PDX1+ (“double positive” or DP) (4, 6.9%), and ARX-negative/PDX1-negative (“double negative” or DN) (32, 55.2.%). Following the methodology of Cejas and Hackeng (12, 40), the ARX-positive/PDX1-negative and DN groups were merged into a single group (ARX++DN), comprising 36 cases (62.1.%). Similarly, the ARX-negative/PDX1-positive and double-positive cases were combined into the PDX1++DP group, totaling 22 cases (37.9.%).

**Table 4:** Comparison of clinical and pathological features based on different expression combinations of ATRX/DAXX and ARX/PDX1 in 58 primary pancreatic neuroendocrine tumors.

Clinicopathological characteristics	ATRX and DAXX positive n=7 (12.1%)	ATRX or DAXX loss n= 51 (87.9%)	P value	ARX++DN n=36 (62.1%)	PDX1++DP n=22 (37.9%)	P value
Age at diagnosis, (range), years	48 (19,72)	61 (22,82)	0.248	61.5 (22,82)	49.5 (19,78)	0.496
Gender, n (%)						
Female	4 (57.14%)	30 (58.82%)	1.000	19 (52.78%)	15 (68.18%)	0.248
Male	3 (42.86%)	21 (41.13%)		17 (47.22%)	7 (31.82%)	
Mean tumor size (range), cm	2.2 (0.8,2.6)	2.6 (0.3,9)	0.211	2.8 (0.3, 7.3)	2.45 (0.4,9)	0.422
Tumor location, n (%)						
Head, neck and uncinate	3 (42.86%)	10 (19.61%)	0.180	6 (16.67%)	7 (31.82%)	0.179
Body-tail	4 (57.14%)	41(80.39%)		30 (83.33%)	15 (68.18%)	
Tumor secretion, n (%)						
Functional	4 (57.1%)	7 (13.7%)	*0.02*	4 (11.11%)	7 (31.82%)	0.083
Nonfunctional	3 (42.9%)	44 (86.3%)		32 (88.89%)	15 (68.18%)	
Tumor genetics, n (%)						
Sporadic	5 (71.43%)	49 (96.08%)	0.067	34 (94.44%)	20 (90.91%)	0.630
Familial	2 (28.57%)	2 (3.92%)		2 (5.56%)	2 (9.09%)	
WHO grade, n (%)						
G1	3 (42.86%)	32 (62.75%)	0.418	22 (61.11%)	13 (59.09%)	0.879
G2	4 (57.14%)	19 (37.25%)		14 (38.89%)	9 (40.91%)	
Lymphovascular invasion, n (%)						
Absent	6 (85.71%)	35 (68.63%)	0.661	25 (69.44%)	16 (72.73%)	0.790
Present	1 (14.29%)	16(31.37%)		11 (30.56%)	6 (27.27%)	
Perineural invasion, n (%)						
Absent	6 (85.71%)	46 (90.20%)	0.555	33 (91.67%)	19 (86.36%)	0.664
Present	1 (14.29%)	5 (9.80%)		3 (8.33%)	3 (13.64%)	
pTumor stage, n (%)						
T1	3 (42.86%)	22 (43.14%)	1.000	15 (41.67%)	10 (45.45%)	0.777
T2-T4	4 (57.14%)	29 (56.86%)		21 (58.33%)	12 (54.55)	
Regional node (pN) stage, n (%)						
N0	6 (85.71%)	40 (78.43%)	1.000	28 (77.78%)	18 (81.82%)	1.000
N1	1 (14.29%)	11 (21.57%)		8 (22.22%)	4 (18.18%)	
Metachronous metastases, n (%)						
Present	2 (28.57%)	13 (25.49%)	1.000	10 (27.78%)	5 (22.73%)	0.670
Absent	5 (71.43%)	38 (74.51%)		26 (72.22%)	17 (77.27%)	
Synchronous metastases, n (%)						
Present	0 (0%)	8 (15.69%)	0.577	5 (13.89%)	3 (13.64%)	1.000
Absent	7 (100%)	43 (84.31%)		31 (86.11%)	19 (86.36%)	
Tumor size, n (%)						
<2cm	2 (28.57%)	17 (33.33%)	1.000	13 (36.11%)	9 (40.91%)	0.715
≥ 2cm	5 (71.43%)	34 (66.67%)		23 (63.89%)	13 (59.09%)	
Surgery, n (%)						
R0	7 (100%)	44 (86.27%)	0.581	32 (88.89%)	19 (86.36%)	1.000
R1	0 (0%)	7 (13.73%)		4 (11.11%)	3 (13.64%)	
Locoregional recurrence, n (%)						
Absent	6 (85.71%)	47 (92.16%)	0.487	33 (91.67%)	20 (90.91%)	1.000
Presence	1 (14.29%)	4 (7.84%)		3 (8.33%)	2 (9.09%)	
Recurrence, n (%)						
Absent	5 (71.43%)	25 (49.02%)	0.425	17 (47.22%)	13 (59.09%)	0.380
Present	2 (28.57%)	26 (50.98%)		19 (52.78%)	9 (40.91%)	
Overall survival (OS) (range), months	19 (3,216)	16 (3,259)	0.623	21 (3,259)	4.5 (3,73)	0.039
Recurrence free survival (RFS), (range), months	19 (3,156)	9 (3,120)	0.335	13 (3,156)	4.5 (3,73)	0.082

DN or “ double negative”, DP or “double positive”.

Results are reported as n (%) or median (min–max) as appropriate. p-values were determined using Pearson’s chi-square or Fisher’s exact test for categorical variables, and the Mann–Whitney U test for continuous variables. Statistically significant differences are highlighted in bold font.

According to the literature (12, 40), we further categorized our cohort into two groups: one defined as negative for ATRX and/or DAXX (indicating loss of at least one marker), and the other comprising cases with preserved expression of both ATRX and DAXX. Additionally, the loss of either ATRX or DAXX was detected in 51 cases (87.9%), including isolated ATRX loss (n=3), isolated DAXX loss (n=4), and concurrent loss of both markers (n=44). Co-expression of both proteins was observed in 7 cases (12.1%).

Among tumors with ATRX/DAXX loss, 44 (86.3%) were NF-PanNETs and 7 (13.7%) were functional (F) PanNETs (χ² = 7.550, p = 0.02).

### 3.6 Immunohistochemical analysis of quantitative ARX, PDX1, ATRX/DAXX, SSTR2a, and SSTR5 expression

The quantitative analysis of ARX, PDX1, ATRX/DAXX, SSTR2a, and SSTR5 expression was performed to determine their sensitivity and specificity concerning clinico-pathological parameters. However, the absence of statistically significant ROC curves impeded the determination of definitive cutoff values for these immunohistochemical markers.

**Figure 2:**
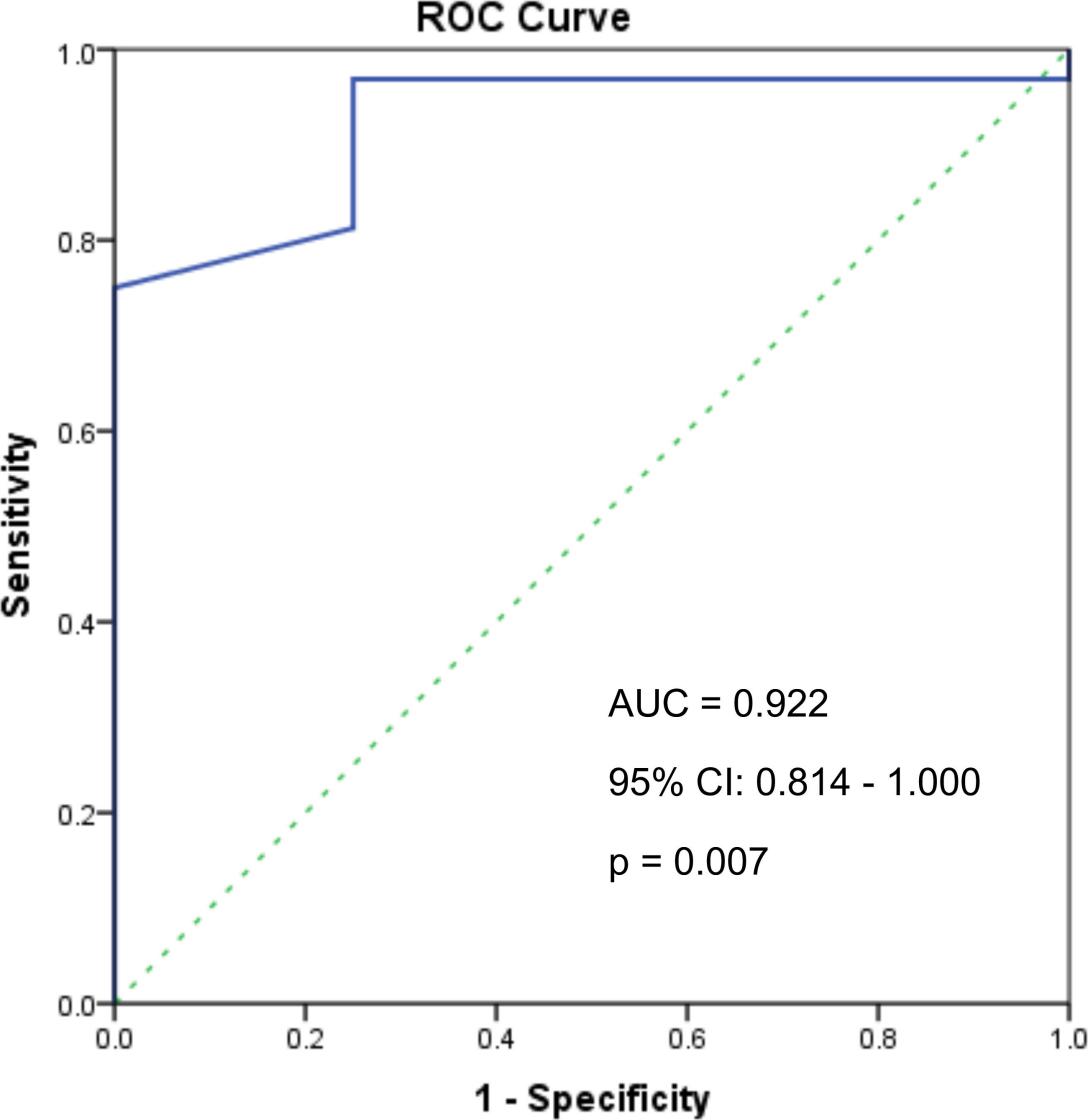
ROC curve analysis of tumor size as a predictor of ATRX/DAXX mutations in pancreatic neuroendocrine tumors ≥ 2 cm (n=36).

**Figure 3:**
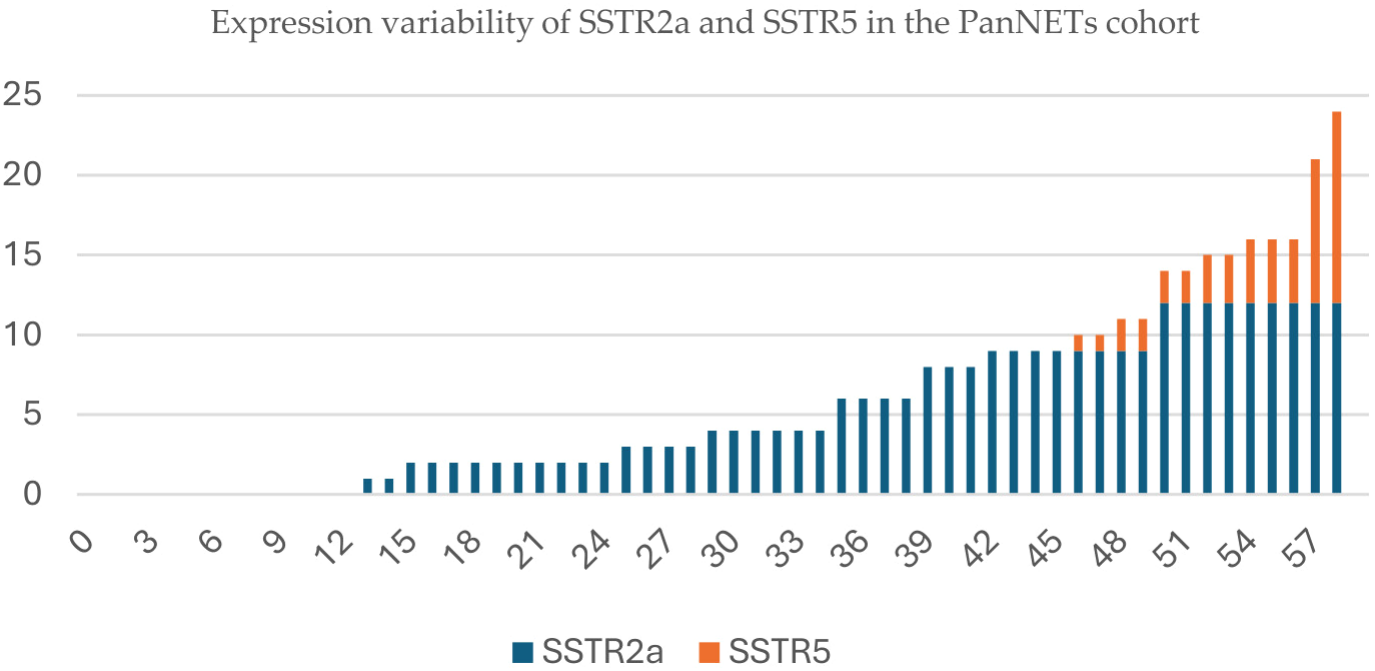
The expression levels of SSTR2a (blue) and SSTR5 (orange) across the PanNETs cohort based on immunoreactivity score.

Still, to assess the impact of tumor size in relation to the loss of ATRX/DAXX protein expression ATRX/DAXX, a ROC analysis was performed on a subset of 36 PanNET cases with tumors measuring ≥ 2 cm. Additionally, a power analysis using the MedCalc Power Estimator for the ROC curve in **Figure 2** demonstrated a statistical power of 84.1% (42). ROC analysis revealed that a cutoff of 2.45 cm was associated with ATRX/DAXX loss ATRX/DAXX at diagnosis, with a 96.9% sensitivity and a 75% specificity (AUC: 0.922, 95% CI: 0.814–1.000, p = 0.007; Youden’s Index = 71.9%) (**Figure 2**).Furthermore, a waterfall plot (**Figure 3**) was used to illustrate the variability in the quantitative SSTR2a and SSTR5 expression based on IRS score within the PanNETs cohort, enabling a comparative analysis of individual marker expression profiles.

### 3.7 The prognostic significance of ARX, PDX1, ATRX/DAXX and SSTR2a and 5 in PanNETs

Using nonparametric comparisons (Mann–Whitney U test), patients with PanNETs who developed metachronous metastases had higher median OS than those without metastases (29 months [range: 3–259] versus 6 months [range: 3–84], p = 0.015). Similarly, patients receiving SSAs therapy (n = 12, 20.7%) showed higher median OS (25 months [range: 11–216] vs. 6 months [range: 3–259], p = 0.05) and RFS (20 months [range: 9–156] vs. 5 months [range: 3–120], p = 0.04) compared to untreated patients.

SSTR5-positive tumors were also associated with higher median OS (73 months [range: 33–216] vs. 7 months [range: 3–259], p < 0.001) and longer median RFS (36 months [range: 14–156] vs. 6 months [range: 3–120], p = 0.005). This pattern was maintained in the subgroup of NF- PanNETs without synchronous metastases, where SSTR5-positive patients showed higher median OS (73 months [range: 46–84] vs. 6 months [range: 3–72], p = 0.02).

.PanNETs patients with PDX1- or ARX++DN tumors had a higher median OS of 21 months [range: 3–259] compared to 4.5 months [range: 3–73] for PDX1+ or PDX1++DP (p = 0.039). Patients with metastatic PanNETs (both synchronous and metachronous) with ARX++DN tumors had a higher median OS compared to PDX1++DP tumors (33 months [range: 3–259] versus 13.5 months [range: 3–29], p = 0.006).

**Figure 4:**
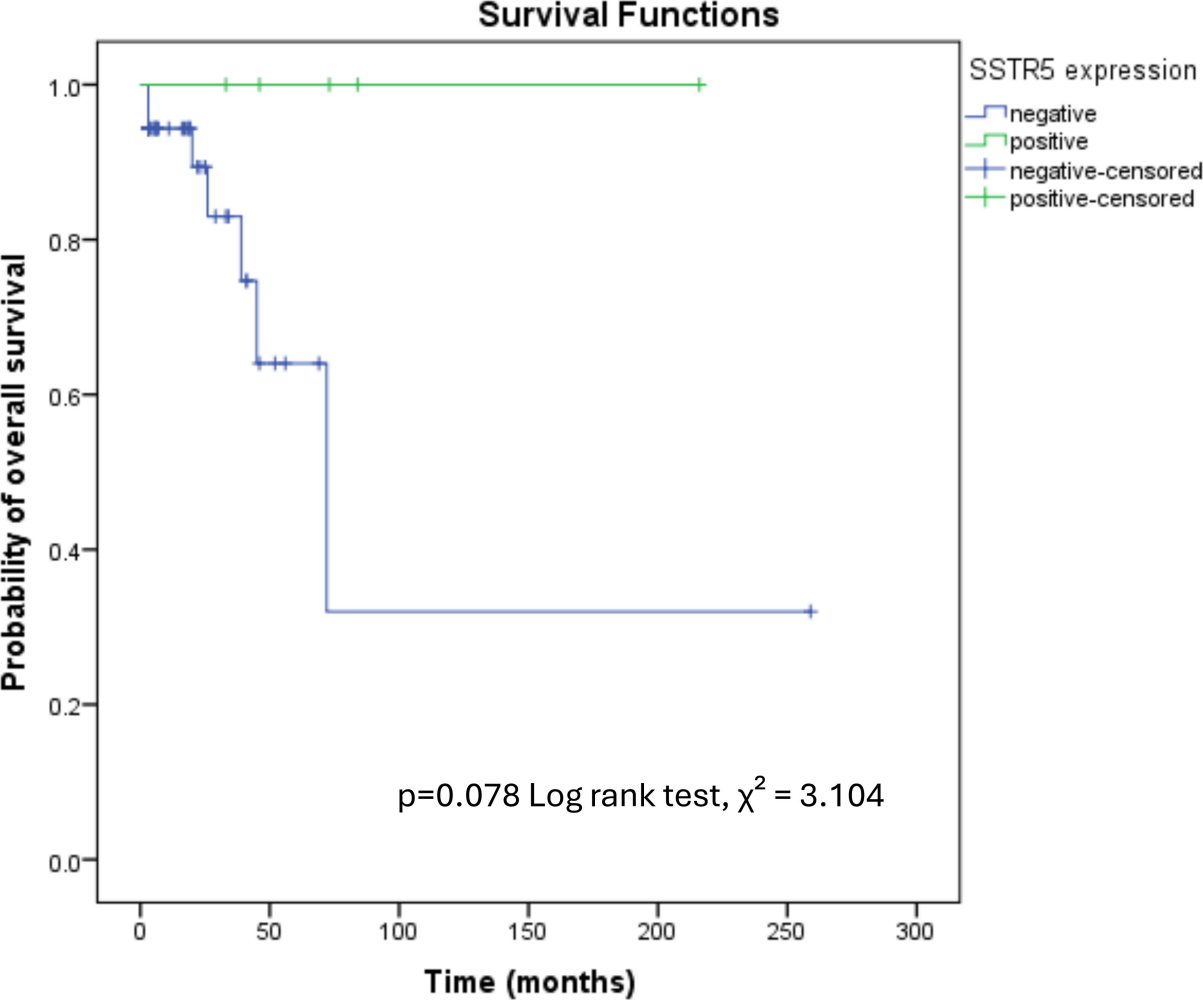
Kaplan–Meier overall survival curves according to SSTR5 expression in PanNET patients (n = 58). A trend toward longer survival was observed in SSTR5-positive cases, without statistical significance (log-rank p = 0.078).

However, Kaplan-Meier analyses of RFS, CSS, and OS demonstrated no statistically significant differences based on ATRX, DAXX, ARX, PDX1, SSTR2a, SSTR5 expression, nor their combinations. Consistently, although median OS was higher in SSTR5-positive cases (73 vs. 7 months), this difference did not reach statistical significance in Kaplan–Meier analysis (log-rank p = 0.078, **Figure 4**). A trend toward longer survival in SSTR5-positive tumors was observed; however, the SSTR5-positive subgroup was small (n = 5), and no events occurred, limiting the robustness of the survival estimates.

Similar results were obtained when restricting the analysis to patients with NF-PanNETs without synchronous metastases, with no statistically significant differences observed. In a subgroup analysis including only patients who did not receive SSA therapy, Kaplan–Meier analysis showed no significant differences in OS between SSTR-positive and SSTR-negative tumors SSTR5: p = 0.238, log-rank test, χ² = 1.395; SSTR2: p = 0.359, log-rank test, χ² = 0.840) or RFS (SSTR5: p = 0.631, log-rank test, χ² =0.230; SSTR2: p = 0.954, log-rank test, χ²=0.003). Cox regression analysis in this subgroup confirmed that SSTR expression was not associated with OS (SSTR5: HR = 30.3, p = 0.468; SSTR2: HR = 1.98, p = 0.373) or RFS (SSTR5: HR = 1.62, p = 0.644; SSTR2: HR = 0.97, p = 0.956).

Furthermore, univariate and multivariate Cox regression analyses showed that neither the investigated biomarkers, nor the established clinicopathological parameters were significantly associated with OS, RFS, or CSS. Additional analyses of quantitative biomarker expression levels did not reveal any statistically significant associations with survival outcomes, and no reliable cutoff values could be established.

## 4 Discussion

We conducted a comprehensive analysis of the individual and combined expression patterns of ARX, PDX1, ATRX/DAXX, and SSTR2a and SSTR5 using IHC on resected tumor specimens to evaluate their impact on the primary endpoint, specifically their correlations with standard clinicopathological factors in primary PanNETs. Additionally, for the secondary endpoint, we investigated the prognostic significance of these biomarkers in relation to clinical outcomes, including the RFS, CSS, and OS.

In Europe, the PanNET incidence is estimated at 1.33-2.33 cases per 100,000 inhabitants (44), with PanNETs comprising 11% of all NEN cases (6). Epidemiological studies link this rise to improved diagnostic tools, particularly in endoscopy and nuclear medicine, leading to the detection of more cases during early, asymptomatic stages. While they can occur at any age, PanNETs are most commonly diagnosed at a mean age at diagnosis of 57 years, and there is a higher prevalence in males (45). Consistent with prior reports, our cohort’s epidemiological features included a median age at diagnosis of 59 years (range 18–82), a predominance of NF-PanNETs (81%), a higher incidence of G1 tumors (60.3%) with T1 stages (43%), but with a slight female predominance (58.6%).

The latest WHO classification of NENs (1) enables better tumor stratification and highlights the importance of immunohistochemical staining for SSTR2a, SSTR5, Rb, p53, ATRX, and DAXX in distinguishing G3 NETs from NECs, including PanNENs and facilitating molecular targeting and therapeutic assessment. Profiling SSTR2/5, along with ARX, PDX1, and ATRX/DAXX, may help predict the efficacy of SSAs and improve PanNETs stratification.

The significant expression of SSTRs occurs in 80–90% of NEN cases, with five subtypes (SSTR1–5) identified (46). The SSTR2 subtype is the most frequently detected, in more than 80% of PanNETs, and it is mainly located in the α- and β-cells of the pancreas, whereas SSTR5 is predominantly expressed in α-cells (47, 48). Our findings are consistent with those of other studies (49) showing a high overall positivity for SSTR2a (53.4% for SSTR2a) in our cohort.

Current literature reports that SSTR2 and SSTR5 are associated with improved survival outcomes (20, 50, 51). Still, the prognostic value of SSTR5 remains unclear, with some studies showing no association between SSTR5 expression and survival endpoints (52, 53).

Considering the reported favorable prognostic role of SSTR2a, we analyzed its expression after excluding PanNETs with synchronous metastases. Negative SSTR2a or SSTR5 expression was more frequent in patients with metachronous metastases compared to those positive for both markers (p = 0.038). SSTR2a expression was also more common in tumors without lymphovascular invasion (p = 0.041), consistent with its association with less aggressive behavior (19).

In line with other studies (46), SSTR5 positive tumors exhibited higher median OS (73 vs. 7 months, p < 0.01) and RFS (36 vs. 6 months, p = 0.005) compared to SSTR5 negative tumors. However, these findings were not confirmed in survival analyses accounting for censoring, with no statistically significant difference in OS (p = 0.078), possibly due to the limited sample size and differences in analytical approach.

Similarly, subgroup analyses restricted to patients without SSA treatment in the context of prognostic evaluation of SSTR expression, as well as to NF-PanNETs without synchronous metastases, yielded consistent results, with no significant survival differences identified. These findings were further supported by Cox regression models, which did not demonstrate independent associations between the investigated biomarkers and survival outcomes.

Taken together, our results suggest that SSTR expression, particularly SSTR5, does not represent a reliable independent prognostic factor in this cohort. Instead, its clinical relevance may be more closely related to its role in therapeutic stratification rather than intrinsic tumor biology. Further studies in larger, independent cohorts are warranted to better clarify the prognostic versus predictive role of SSTR expression.

Furthermore, we found no association between SSTR2a or 5 expression and tumor location, functional status, or TNM staging, in line with previous studies (48, 54). Differences may stem from our cohort’s distribution, which included more stage 1–3 tumors than stage 4 tumors, and more nonfunctional than secretory PanNETs.

Although the association between SSTR expression and survival varies between studies, SSTR2 and SSTR5 positivity generally indicate a better prognosis (19, 53, 54). This may be also related to treatment with SSAs or to the pro-apoptotic effects of somatostatin signaling. Consistent with this hypothesis, in our cohort patients treated with SSAs (which are associated with SSTR positivity) had a longer median OS (25 months [range: 11–216] vs. 6 months [range: 3–259], p = 0.05) and a longer RFS (20 months [range 9–156] versus 5 months [range: 3–120], p = 0.04) compared to untreated patients. Establishing robust genotype–phenotype associations in PanNETs could facilitate the routine use of IHC in clinical practice. IHC for DAXX shows an 85% sensitivity and a 95% specificity, although the performance varies depending on the mutation site (21). While early studies linked ATRX/DAXX loss to improved outcomes, subsequent data have associated this finding- often indicative of ALT activation- with reduced RFS (22, 55). Discrepancies between studies suggest that ATRX loss correlates with a shorter OS; however, it may still represent a less reliable prognostic marker compared to loss of DAXX immunoreactivity (56).

The reported frequency of ATRX/DAXX mutations ranges from 12% to 40% (34), however, we observed loss of protein expression in 87.9% of cases, which is comparable to the 79% reported by Park et al. (29). This discrepancy is likely due to differences in the IHC assessment methods. Consistent with the findings reported in the literature (12), ATRX/DAXX loss was observed in 44 NF-PanNETs and 7 F-PanNETs(p = 0.02). Additionally, ATRX-negative tumors were more commonly located in the body or tail of the pancreas than in the head, neck, or uncinate process (p = 0.042).Discrepancies between studies may also result from differences in cohort composition: while Jiao et al. and Raj et al. found ATRX/DAXX mutations to be favorable prognostic markers in metastatic populations (57, 58), Chou et al. (59) reported the opposite in a cohort with only 13% metastatic cases. A recent meta-analysis showed a non-significant trend toward longer survival in ATRX/DAXX-altered cases (p = 0.96) (34). In our cohort, which included 13.8% of patients with synchronous metastases, there were no significant differences in the survival after the exclusion of this variable. Accumulating evidence indicates that DAXX and ATRX mutations emerge in advanced PanNETs following the progression of initial microadenomas to larger tumors exceeding 3 cm, which is consistent with our findings (22, 60). Our ROC analysis assessed the relationship between tumor size and loss of ATRX/DAXX protein expression in a subset of 36 PanNET cases with tumors ≥ 2 cm. A tumor size cutoff of 2.45 cm was associated with loss of ATRX/DAXX protein expression at diagnosis, with a sensitivity of 96.9% and a specificity of 75% (AUC 0.922, p = 0.007), demonstrating good discriminatory performance. However, given the relatively small sample size (n = 36), this finding should be considered hypothesis-generating and requires validation in larger, prospective, multi-institutional cohorts before clinical application.

Additionally, recent investigations have demonstrated a higher prevalence of DAXX and ATRX loss in metastatic lesions (approximately 62%) compared to primary tumors (around 25%). This disparity suggests a strong association between the loss of these proteins and tumor progression, underscoring the importance of elucidating the underlying molecular mechanisms driving this phenomenon (12).

These findings suggest that ATRX/DAXX loss may be associated with more aggressive disease and is linked to later-stage tumors (12, 22, 23, 61, 62), warranting a repeat biopsy in cases of tumor progression.

ARX expression can be reliably assessed using cytological samples with a low interobserver variability, although false-positive results for PDX1 may occur, potentially attributed to contamination or sampling issues (39). In our study, the proportions of ARX+, PDX1+, DP, and DN tumors were 13.8%, 37.9%, 6.9%, and 55.2%, respectively—substantially different from the proportions reported by Cejas et al. (40)., which were 43%, 19%, 28%, and 10%.

While the ARX expression was correlated with adverse prognostic features in an international cohort, it showed no association with the RFS (12). The lower rate of ARX+ tumors compared to the 50–60% reported in recent studies (40, 63) may have been due to selection bias and the more indolent behavior of the disease in our cohort. This observation is corroborated by the fact that ARX++DN tumors had longer median survival than other subgroups (21 months [range: 3–259] vs. 4.5 months [range: 3-73], p = 0.039)[65]. The median OS in metastatic (both synchronous and metachronous) ARX++DN tumors was 33 months [range:3-259] compared to 13.5 months [range: 3–29] in PDX1++DP tumors (p = 0.006), also suggesting a more indolent disease course.

The ARX++DN profile in NF-PanNETs has been associated with aggressive features a higher incidence of locoregional recurrences and distant metastases, particularly in MEN1 patients (12, 40). The lack of significance in our study may have been due to the limited number of MEN1 cases, which may have prevented the detection of significant associations with the tumor size, grade, and lymph node involvement.

In summary, we established a Romanian cohort of PanNET cases to characterize their clinicopathological and molecular features through immunohistochemical analysis. Our findings align with the current literature, indicating that positive expression of SSTR5 is associated with better median OS and RFS. Additionally, our findings suggest that within the subgroup of PanNETs ≥ 2 cm, the tumor size, specifically a cutoff of ≥ 2.45 cm, may be associated with the loss of ATRX/DAXX protein expression in larger tumors. ATRX/DAXX. These insights support the growing evidence that the ATRX/DAXX and SSTR5 status is a critical factor in the biological behavior and clinical management of PanNETs. In contrast, ARX/PDX1 results were inconclusive, likely due to the small number of ARX-positive cases. These findings highlight the heterogeneity of PanNETs and the need for further investigation, particularly regarding metastatic progression. The present study faced several limitations. First, the retrospective design and the heterogeneous biological behavior of the disease could have introduced potential bias. During the treatment period of our cohort, which was over 20 years, the patient outcomes might have been influenced by significant advancements in surgical resection and systemic treatments. The analysis might have been further influenced by the use of categorical variable boundaries applied to continuous data. The relatively small cohort size and short follow-up (median: 16.5 months) may have affected the stability, robustness, and generalizability of the findings, including those derived from both survival and ROC analyses. Additionally, the absence of correction for multiple testing and may increase the risk of Type I error.

. Additionally, in cases of surgical interventions such as enucleation, resulting in a suboptimal lymphadenectomy, lymph node metastases may have been underestimated. In future analyses, we aim to expand our cohort and examine the correlation between tumor specimens and an FNAB samples to improve risk stratification. Additional improvements could be obtained by using IHC for ATRX/DAXX to distinguish between NETs and NECs, and to identify the primary site in cases of metastatic NETs. Furthermore, prognostic assessment could be enhanced by integrating ALT status (via FISH) with ATRX/DAXX expression. These future results could be representative of the Romanian population, representing a step toward future large prospective studies to confirm the effectiveness and generalizability of these biomarkers.

## 5 Conclusions

In conclusion, the evaluation of ATRX, DAXX, ARX, PDX1, and SSTR2a/5 protein expression is cost-effective and rapid, offering an opportunity to positively influence the decision-making process. Refining the classification of PanNENs into distinct tumor subtypes based on a molecular signature through an immunohistochemical evaluation of the aforementioned biomarkers could enhance the prognostic accuracy and optimize patient management.

## Data Availability

The original contributions presented in the study are included in the article/**Supplementary Material**. Further inquiries can be directed to the corresponding authors.
