# Supplementary material for: Expression of ATRX, DAXX, PDX1, ARX, and somatostatin receptors in pancreatic neuroendocrine tumors: a clinicopathological study
Source: Front Endocrinol (Lausanne). 2026 May 11;17:1820433. doi: 10.3389/fendo.2026.1820433 (PMC13199031; doi:10.3389/fendo.2026.1820433)
Supplement: Supplementary file 1 [file Table1.docx]

|  | SSTR2a negative  n= 27 (46.6%) | SSTR2a positive  n=31 (53.4%) | P value | SSTR5 negative  n=53 (91.4%) | SSTR5 positive  n= 5 (8.6%) | | P value | |  | |
| --- | --- | --- | --- | --- | --- | --- | --- | --- | --- | --- |
| Age at diagnosis, range, years | 54 (19,82) | 59 (22,78) | 0.374 | 59 (19,82) | | 56 (27,67) | | 0.872 | |  |
| Gender, n (%) |  |  |  |  | |  | |  | |  |
| Female | 17 (62.96%) | 17 (54.84%) | 0.531 | 33 (62.26%) | | 1 (20.00%) | | 0.149 | |  |
| Male | 10 (37.04%) | 14 (45.16%) |  | 20 (37.74%) | | 4 (80.00%) | |  | |  |
| Mean tumor size, range, cm | 2.5 (0.4, 7.3) | 2.5 (0.3,9) | 0.580 | 2.5 (0.3,9) | | 2.5 (2.2, 7.3) | | 0.433 | |  |
| Tumor location, n (%) |  |  |  |  | |  | |  | |  |
| Head, neck and uncinate | 5 (18.52%) | 8 (25.81%) | 0.507 | 11 (20.75%) | | 2 (40.00%) | | 0.311 | |  |
| Body-tail | 22 (81.48%) | 23 (74.19%) |  | 42 (79.25%) | | 3 (60.00%) | |  | |  |
| Tumor secretion, n (%) |  |  |  |  | |  | |  | |  |
| Functional | 8 (29.63%) | 3 (9.68%) | 0.091 | 9 (16.98%) | | 2 (40.00%) | | 0.237 | |  |
| Nonfunctional | 19 (70.37%) | 28 (90.32%) |  | 44 (83.02%) | | 3 (60.00%) | |  | |  |
| Tumor genetics, n (%) |  |  |  |  | |  | |  | |  |
| Sporadic | 24 (88.89%) | 30 (96.77%) | 0.329 | 50 (94.34%) | | 4 (80.00%) | | 0.310 | |  |
| Familial | 3 (11.11%) | 1 (3.23%) |  | 3 (5.66%) | | 1 (20.00%) | |  | |  |
| WHO classification, n (%) |  |  |  |  | |  | |  | |  |
| G1 | 18 (66.67%) | 17 (54.84%) | 0.358 | 33 (62.26%) | | 2 (40.00%) | | 0.376 | |  |
| G2 | 9 (33.33%) | 14 (45.16%) |  | 20 (37.74%) | | 3 (60.00%) | |  | |  |
| Lymphovascular invasion, n (%) |  |  |  |  | |  | |  | |  |
| Absent | 23 (85.19%) | 18 (58.06%) | ***0.041*** | 37 (69.81%) | | 4 (80.00%) | | 1.000 | |  |
| Present | 4 (14.81%) | 13 (41.94%) |  | 16 (30.19%) | | 1 (20.00%) | |  | |  |
| Perineural invasion, n (%) |  |  |  |  | |  | |  | |  |
| Absent | 25 (92.59%) | 27 (87.10%) | 0.675 | 47 (88.68%) | | 5 (100.00%) | | 1.000 | |  |
| Present | 2 (7.41%) | 4 (12.90%) |  | 6 (11.32%) | | 0 (0.00%) | |  | |  |
| pTumor stage (pT), n (%) |  |  |  |  | |  | |  | |  |
| T1 | 13 (48.15%) | 12 (38.71%) | 0.469 | 23 (43.40%) | | 2 (40.00%) | | 1.000 | |  |
| T2-T3-T4 | 14 (51.85%) | 19 (61.29%) |  | 30 (56.60%) | | 3 (60.00%) | |  | |  |
| Lymph node status (pN) , n (%) |  |  |  |  | |  | |  | |  |
| N0 | 25 (92.59%) | 21 (67.74%) | ***0.025*** | 42 (79.25%) | | 4 (80.00%) | | 1.000 | |  |
| N1 | 5 (18.52%) | 10 (32.26%) |  | 11 (20.75%) | | 1 (20.00%) | |  | |  |
| Metachronous metastases, n (%) |  |  |  |  | |  | |  | |  |
| Present | 7 (25.93%) | 8 (25.81%) |  | 12 (22.64%) | | 3 (60.00%) | | 0.103 | |  |
| Absent | 20 (74.07%) | 23 (74.19%) |  | 41 (77.36%) | | 2 (40.00%) | |  | |  |
| Synchronous metastases, n (%) |  |  |  |  | |  | |  | |  |
| Present | 2 (7.41%) | 6 (19.35%) |  | 8 (15.09%) | | 0 (0.00%) | | 1.000 | |  |
| Absent | 25 (92.59%) | 25 (80.65%) |  | 45 (84.91%) | | 5 (100.00%) | |  | |  |
| Tumor size, n (%) |  |  |  |  | |  | |  | |  |
| <2cm | 12 (44.44%) | 10 (32.26%) | 0.340 | 31 (58.49%) | | 5 (100.00%) | | 0.145 | |  |
| ≥ 2cm | 15 (55.56%) | 21 (67.74%) |  | 7 (13.21%) | | 0 (0.00%) | |  | |  |
| Surgery, n (%) |  |  |  |  | |  | |  | |  |
| R0 | 26 (96.30%) | 25 (80.65%) | 0.108 | 46 (86.79%) | | 5 (100.00%) | | 1.000 | |  |
| R1 | 1 (3.70%) | 6 (19.35%) |  | 7 (13.21%) | | 0 (0.00%) | |  | |  |
| Locoregional recurrence, n (%) |  |  |  |  | |  | |  | |  |
| Absent | 24 (88.89%) | 29 (93.55%) | 0.656 | 49 (92.45%) | | 4 (80.00%) | | 0.374 | |  |
| Present | 3 (11.11%) | 2 (6.45%) |  | 4 (7.55%) | | 1 (20.00%) | |  | |  |
| Recurrence, n (%) |  |  |  |  | |  | |  | |  |
| Absent | 14 (51.85%) | 16 (51.61%) | 0.986 | 29 (54.72%) | | 1 (20.00%) | | 0.187 | |  |
| Present | 13 (48.15%) | 15 (48.39%) |  | 24 (45.28%) | | 4 (80.00%) | |  | |  |
| Overall survival (OS), range, months | 19 (3,259) | 11 (3,216) | 0.892 | 7 (3,259) | | 73 (33,216) | | ***< 0.001*** | |  |
| Recurrence free survival (RFS) range, months | 6 (3,120) | 6 (3,156) | 0.842 | 6 (3,120) | | 36 (14,156) | | 0.005 | |  |

**Table 1. Clinical and pathological comparison of SSTR2a and SSTR5 expressions in 58 primary pancreatic neuroendocrine tumors.**

*Results are reported as n (%) or median (min–max) as appropriate. p-values were determined using Pearson's chi-square or Fisher’s exact test for categorical variables, and the Mann–Whitney U test for continuous variables. Statistically significant differences are highlighted in bold font.*
