# Supplementary material for: Expression of ATRX, DAXX, PDX1, ARX, and somatostatin receptors in pancreatic neuroendocrine tumors: a clinicopathological study
Source: Front Endocrinol (Lausanne). 2026 May 11;17:1820433. doi: 10.3389/fendo.2026.1820433 (PMC13199031; doi:10.3389/fendo.2026.1820433)
Supplement: Supplementary file 2 [file DataSheet1.pdf]

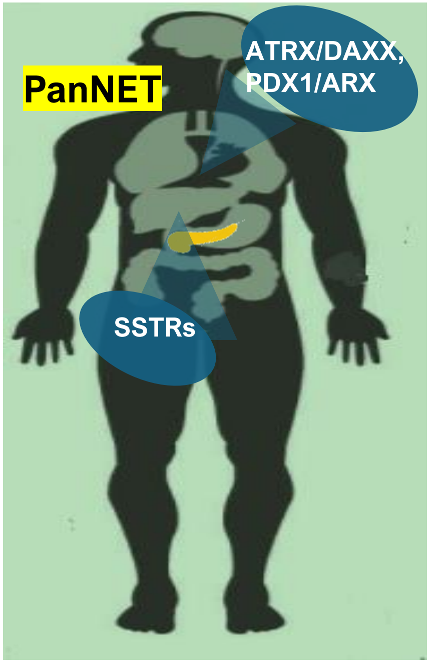

DAXX/ATRX and PDX1/ARX as potential prognostic biomarkers defining new PanNETS subtypes

The role of SSTR subtype expression in PanNETs and its clinical relevance remains unclear.

Retrospective study

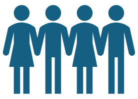

58 patients with PanNENs

Immunohistochemical analysis

Dichotomized analysis of ATRX, DAXX, ARX/PDX1, and SSTR2a/5.

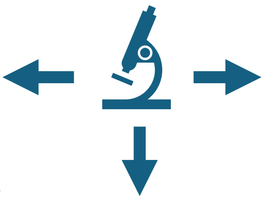

Quantitative analysis of the expression of these proteins

ROC anc Kaplan-Meier curve analysis

ROC curve for the predictive value of DAXX and ATRX expression for identifying sporadic cases in PanNETs ≥ 2 cm

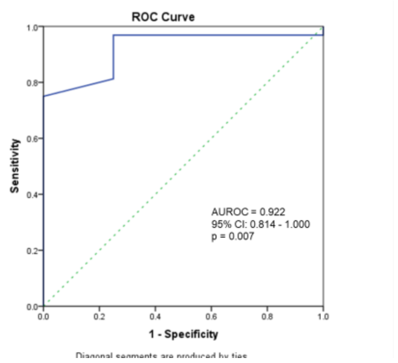

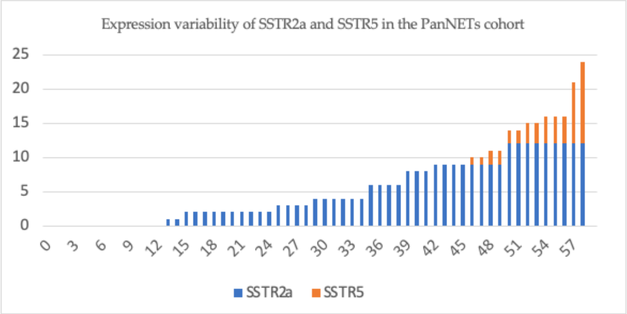

Patients with SSTR5+ tumors had a longer median OS time (73 months [range 33-216] versus 7 months [range 3-216], p <0.001). Patients with SSTR5+ tumors had a longer median RFS time (36 months [range 14-156] versus 6 months [range 3-120], p =0.005).

DAXX/ATRX mutations → advanced PanNETs

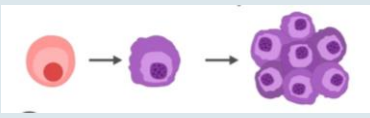

Mortality 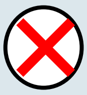 SSTR5 ↓ 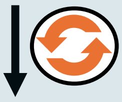 **Recurrence**

DAXX/ATRX mutations, which occur in larger tumors, are associated with disease progression, while higher SSTR5 levels are indicative of a better prognosis in patients with PanNETs.
